# *Spilanthes acmella* Extract-Based Natural Oils Loaded Emulgel for Anti-Microbial Action against Dermatitis

**DOI:** 10.3390/gels9100832

**Published:** 2023-10-20

**Authors:** Aqsa Afzal, Syed Nisar Hussain Shah, Hina Javed, Asma Mumtaz, Javeria Saeed, Hafiz Majid Rasheed, Rabia Arshad, Siddique Akber Ansari, Hamad M. Alkahtani, Irfan Aamer Ansari

**Affiliations:** 1 Department of Pharmaceutics, Faculty of Pharmacy, Bahauddin Zakariya University, Multan 60000, Pakistan; aqsaafzalpharmacist@gmail.com (A.A.); javeriasaeed102@yahoo.com (J.S.); 2 Faculty of Pharmacy, The University of Lahore, Lahore 54590, Pakistan; 3 Department of Pharmaceutical Chemistry, College of Pharmacy, King Saud University, Riyadh 11451, Saudi Arabia; 4 Department of Drug Science and Technology, University of Turin, 10124 Turin, Italy; irfanaamer.ansari@unito.it

**Keywords:** *Spilanthes acmella*, spilanthol, *Staphylococcus aureus*, *Pseudomonas aeruginosa*, *Escherichia coli*, emulgel

## Abstract

Background: Dermatitis is skin disorder that is complicated by recurrent infections of skin by bacteria, viruses, and fungi. Spilanthol is an active constituent of *Spilanthes acmella*, which possess strong anti-bacterial properties. The purpose of this study was to develop a herbal emulgel for the treatment of dermal bacterial infections, as microscopic organisms have created solid resistance against anti-microbials. Methods: Emulgels were prepared and characterized for parameters such as physical examination, rheological studies, spreading coefficient, bio-adhesive strength measurement, extrudability study, antibacterial activity, FTIR analysis, in vitro drug dissolution, and ex vivo permeation studies. Result: With a statistically significant *p*-value = 0.024, 100% antibacterial activity was observed by F4 against *Staphylococcus aureus*, *Pseudomonas aeruginosa*, and *Escherichia coli* (mean ± S.D) (25.33 ± 0.28, 27.33 ± 0.5, and 27 ± 0.5). However, maximum antibacterial effect 100% formulations produced zones of inhibitions against *E. coli* *p*-value = 0.001. The mean zone of inhibition produced by F4 was greatest among all at 26.44 ± 0.37 mm (mean ± S.D). The F4 formulation produced a maximum percentage dissolution, permeation, and flux of 86.35 ± 0.576, 55.29 ± 0.127%, and 0.5532 ug/cm^2^/min, respectively. Conclusions: The present study therefore, suggests the use of *S. acmella* extract and olive oil containing emulgel for treating bacterial skin infections.

## 1. Introduction

Dermatitis is a general term used to describe inflammation of the skin. It can be caused by a variety of factors, such as exposure to irritants or allergens, genetic predisposition, infections, or autoimmune disorders [1]. Conventional therapies for dermatitis typically involve the use of topical or oral medications, as well as lifestyle modifications to reduce exposure to triggers [2]. Some common conventional therapies include topical corticosteroids, immunosuppressive creams, or ointments that can be used as an alternative to corticosteroids [3]. Moreover, antihistamines and antibiotics or antifungal medications may be prescribed in cases of bacterial or fungal infections that are contributing to the dermatitis symptoms [4]. It is important to note that the effectiveness of these conventional therapies can vary depending on the type and severity of the dermatitis [5]. Additionally, the long-term use of some medications, such as corticosteroids, can have side effects, such as skin thinning and increased risks of infection and drug resistance. In the face of this widespread resistance, there is an urgency to produce ways of combating infection without traditional antibiotics. The rapidly emerging field of bioinspired products is one such agent. The use of natural and synthetic biomaterials has the potential to transform conventional therapeutic interventions into novel formulation technologies. Over a thousand years, traditional medicaments and natural products have been used all over the world, and this antecedes the introduction of modern drugs and antibiotics [6]. Therefore, in this study, we preferred using a natural herb-based drug (*Spilanthes acmella*) and a combination of natural oils, including lemon and olive oils, followed by their incorporation into a Carbopol emulgel base. Spilanthol is a natural compound found in the extract of the *Acmella oleracea* plant, also known as the toothache plant. It has been shown to have both antibacterial and anti-inflammatory properties.

The plant has been used to reduce inflammation in various parts of the body, including the mouth and throat. Its anti-inflammatory properties have also made it a component in some traditional remedies for sore throats and mouth ulcers. *Spilanthes acmella* is also believed to have antimicrobial properties and it is used in some cultures to address bacterial or fungal infections. In some traditional systems, *Spilanthes acmella* has been used as an aphrodisiac, possibly due to its ability to stimulate sensations and increase blood flow when consumed. Beyond dental pain, *Spilanthes acmella* has been used in traditional medicine for pain relief in other areas of the body, such as headaches and joint pain. In some culinary traditions, the leaves and flowers of *Spilanthes acmella* are used as a spices or condiments. They have a unique flavor characterized by their tingling, numbing sensation and are sometimes added to salads, soups, or sauces to provide a sensory experience.

As an antibacterial agent, spilanthol has been found to be effective against a range of bacteria, including *Staphylococcus aureus* and *Escherichia coli* [7]. It appears to work by disrupting the bacterial cell membrane, leading to cell death. Spilanthol has been shown to reduce inflammation by inhibiting the production of pro-inflammatory cytokines, such as interleukin-1β and tumor necrosis factor-alpha. This makes it potentially useful in the treatment of conditions characterized by inflammation, such as rheumatoid arthritis, periodontitis, and acne [8,9,10]. Lemon oil possess strong anti-bacterial potential against numerous bacteria, such as *P. aeruginosa*, *E. coli*, and *Staphylococcus aureus*, causing dermatitis [11]. Olive oil, which contains oleuropein and is a phenolic constituent extracted from olives, slows down the growth of *S. aureus* [12]. Lemon and olive oil were also used in the emulgel formulations as a source of permeation enhancement. The purpose was to develop a herbal emulgel formulation to treat skin bacterial manifestations. Emulgels are topical formulations that combine the properties of both an emulsion and a gel. Emulsions are mixtures of oil and water, typically stabilized with an emulsifying agent, while gels are semi-solid systems composed of a network of cross-linked polymers in a liquid base. Emulgels typically have a gel-like consistency, but contain both oil- and water-soluble ingredients, allowing them to deliver a wide range of active ingredients to the skin. Emulgels offer several advantages over other topical formulations, including an improved stability and enhanced skin penetration [13]. The emulsifying agents in the emulgel help to create a stable and uniform dispersion of oil and water, preventing phase separation and ensuring that the active ingredients are evenly distributed throughout the product [13]. The gel-like consistency of the emulgel also allows it to adhere to the skin for longer periods of time, allowing for improved skin penetration and the sustained release of the active ingredients [14]. Emulgels can be used to deliver a wide range of active ingredients, including moisturizers, sunscreens, and anti-inflammatory agents, among others. They are also suitable for use on a variety of skin types, including oily and acne-prone skin, as they are typically non-greasy and absorb quickly [15]. Overall, emulgels are versatile and effective topical formulations that offer a number of advantages over other types of skincare products. Their unique combination of emulsion and gel properties makes them well-suited for a wide range of applications, and they continue to be a popular choice for skincare manufacturers and consumers alike. A syTop of Formnthesized emulgel was characterized for parameters such as physicochemical studies, rheological studies, spreading Coefficient, bio-adhesive strength measurement, extrudability study, antibacterial activity, in vitro drug dissolution, and ex vivo permeation studies [16]. Based on our results, we can conclude that the F4 formulation was the most promising emulgel. The ex vivo skin permeation studies confirmed that our optimized F4 formulation provided the maximum drug release and antibacterial activity.

## 2. Results and Discussion

Natural-oil-loaded emulgels are a type of cosmetic product that combines the benefits of both natural oils and emulsions. Emulgels are a type of gel that contain both water and oil and are stabilized using an emulsifying agent. When natural oils are added to emulgels, they can provide additional nourishing and moisturizing benefits to the skin. The natural oils used in emulgels can vary depending on the desired benefits and properties of the product. For example, argan oil, jojoba oil, and coconut oil are commonly used in skincare products for their moisturizing and nourishing properties. Essential oils, such as lavender and tea tree oil, may also be added for their therapeutic benefits. Emulgels are a popular choice for cosmetic products, because they offer a lightweight, non-greasy texture that is easy to apply and absorbs quickly into the skin. Additionally, emulgels can help to extend the shelf life of natural oils, which can be prone to oxidation and spoilage. Overall, natural-oil-loaded emulgels are a versatile and effective cosmetic product that can provide a wide range of benefits to the skin. Lemon oil and olive oil both have potential antibacterial properties that may be beneficial for dermatitis. Lemon oil contains compounds such as limonene and Citral that have shown antibacterial activity against various bacterial strains. These properties may help to combat bacterial infections that can exacerbate dermatitis symptoms. However, it is important to note that lemon oil is also photosensitizing, which means it can increase the skin’s sensitivity to sunlight and may cause skin irritation in some people. Olive oil also has potential antibacterial properties, particularly against certain strains of Staphylococcus aureus bacteria. This type of bacteria is commonly found on the skin and can contribute to the development of dermatitis. Olive oil also has moisturizing and anti-inflammatory properties that can help to soothe and protect the skin. It is important to note that, while natural oils like lemon oil and olive oil may have antibacterial properties, they should not be relied upon as the sole treatment for dermatitis. In dermatitis, spilanthol may have potential benefits due to its anti-inflammatory properties. Dermatitis is an inflammatory skin condition, characterized by redness, itching, and skin irritation. Spilanthol has been found to inhibit the production of pro-inflammatory cytokines, which are molecules that contribute to inflammation in the body. This suggests that spilanthol may be able to help reduce inflammation and alleviate some of the symptoms of dermatitis. Physically, all emulgels were found to be homogeneous, having no phase separation and grittiness. It has been shown that the viscosity of formulations increases with an increasing conc. of Carbopol 934 [17]. The formulations with the highest viscosity showed a minimum value of spreadability [18]. Formulations with less viscosity had lower values of extrudability [19]. It has been observed that, by increasing concentration of Carbopol, the bio-adhesive strength also increased [19]. Lemon oil was included in the F1 and F2 formulations of emulgel, which itself contains various bioactive constituents like monoterpenes, esters, sesquiterpenes, aldehydes, alcohol, and ketones possessing antibacterial and antifungal properties [20]. Lemon possesses numerous properties like antimicrobial, antifungal, and anti-inflammatory [21]. Olive oil, which was present in our optimized F4 formulation, constitutes polyphenolic and phenolic compounds like hydroxytyrosol and oleuropein that remove free radicals from the body. Olive oil is applied topically to treat various skin diseases. The presence of olive oil in F4 produced the best wound-healing results on the skin [22,23]. Phenolic compounds are important constituents of olive leaves, which possess strong antibacterial potential against various Gram-positive microbes [24]. As it showed 100% antibacterial effect against all formulations, we considered the F4 formulation as an optimized formulation [25,26].

As per our findings, a first-order model was best suited for our emulgels, as the correlation coefficient R^2^ value was close to 1 in first-order kinetics. First-order drug release is dependent upon the concentration of the drug. The F4 formulation contained olive oil, which has been used as a permeation enhancer in various topical formulations, as it has also been reported to provide excellent drug release [22,27].

### 2.1. Physical Appearance of the Emulgels

**Figure 1:**
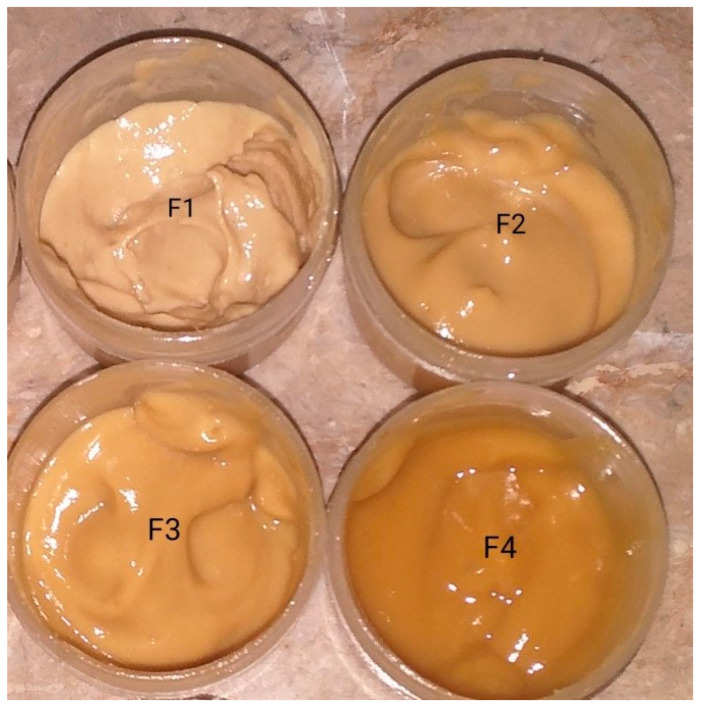
Images of emulgel formulations (F1–F4).

Physically, all the emulgels were found to be homogeneous, having no phase separation and grittiness. Images of the emulgels are shown in Figure 1.

### 2.2. Fourier Transform Infrared Spectroscopy (FTIR)

**Figure 2:**
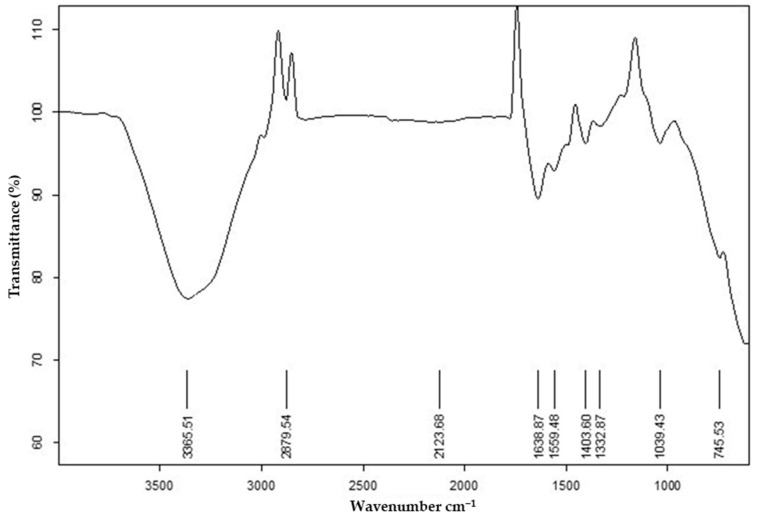
FTIR spectra of F4 formulation.

The FTIR spectrum of the F4 formulation typically exhibits the following peaks. As spilanthol is the active ingredient, its broad peak around 3400–3200 cm^−1^ is attributed to the O-H stretching vibrations of the phenolic hydroxyl group and/or N-H stretching vibrations of the amide group. There is a peak around 2930 cm^−1^, which is assigned to the C-H stretching vibrations of the methyl and methylene groups. A peak around 1700–1650 cm^−1^ is associated with the C=O stretching vibrations of the carbonyl group in the lactone ring. A peak around 1600–1500 cm^−1^ is attributed to the C=C stretching vibrations of the aromatic ring. A peak around 1400 cm^−1^ is assigned to the bending vibrations of the C-H bond in the methyl group. A peak around 1300 cm^−1^ is attributed to the C-O stretching vibrations of the lactone ring and a peak around 1200 cm^−1^ is assigned to the C-O stretching vibrations of the ether linkage, as shown in Figure 2. The FTIR analysis confirmed no evidence of any interaction. The FTIR peaks of the pure extract and formulations (F1–F4) are summarized in Table 1.

### 2.3. Viscosity

**Figure 3:**
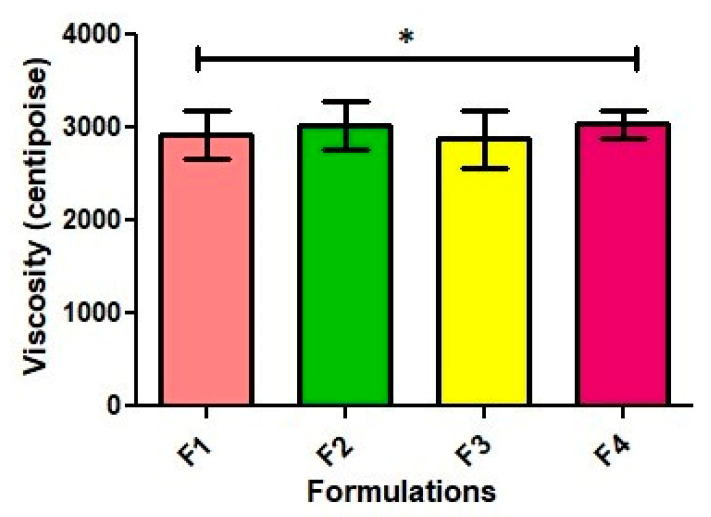
Viscosities of all formulations (centipoise). The results are enumerated as mean ± S.D (*n* = 3). * represents significance level (*p* ≤ 0.05).

An emulgel is a type of topical formulation that combines the properties of both an emulsion and a gel. It typically consists of a water-in-oil or oil-in-water emulsion that is thickened with a gelling agent to form a gel-like consistency. The viscosity of an emulgel can vary depending on the specific formulation and concentration of the gelling agent used. The results showed that the viscosity of the formulations increased with an increasing concentration of Carbopol 934. In general, Carbopol polymer is associated with the provision of a higher viscosity than traditional polymers due to the presence of the emulsion component. The viscosity of an emulgel is an important property to consider in the development and manufacturing of topical formulations, as it can impact the ease of application, the spreadability, and the overall stability of the product. A suitable viscosity can help to ensure that the emulgel is easy to apply and can spread evenly over the skin, while also providing the desired therapeutic effect. The results of all the formulations were found to be in the range from 2870.66 ± 4.91 to 3027 ± 3.05 (Mean ± SEM), as shown in Figure 3.

### 2.4. Spreadability

**Figure 4:**
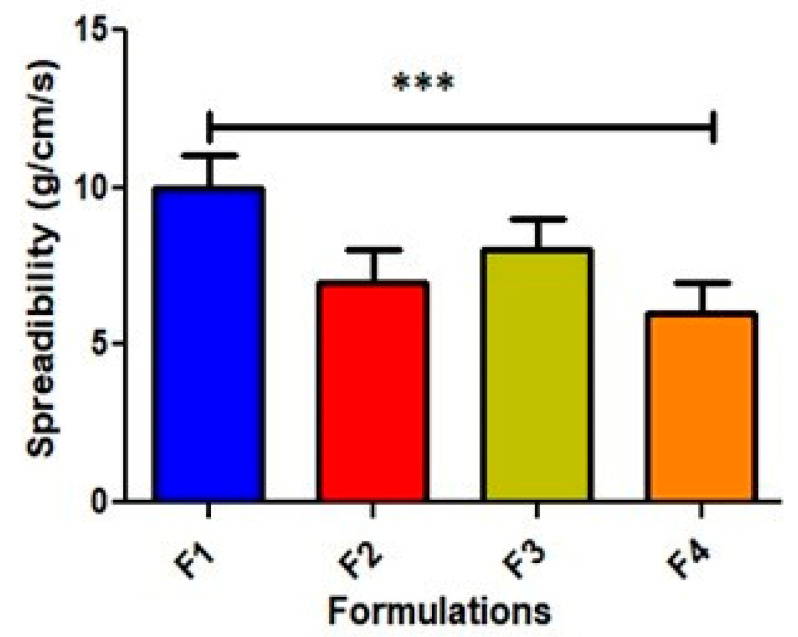
Spreadability of all formulations (g/cm/s). The results are enumerated as mean ± S.D (*n* = 3). *** represents significance level (*p* ≤ 0.001).

The spreadability of an emulgel is an important factor in determining its effectiveness in delivering the active ingredients to the affected area. The spreadability of an emulgel is influenced by several factors, such as the viscosity of the gel, the size and distribution of the emulsion droplets, and the composition of the emulgel. An emulgel with a higher viscosity may be more difficult to spread evenly and may require more force to apply. On the other hand, an emulgel with a lower viscosity may spread too quickly and thinly, making it difficult to deliver an effective dose of the medication. Therefore, the optimum viscosity led to a better spreadability. The results were found in the range of Mean ± SEM from 5.58 ± 0.06 to 8.85 ± 0.39 gm/cm/s, as shown in Figure 4.

### 2.5. Bio-Adhesive Strength

**Figure 5:**
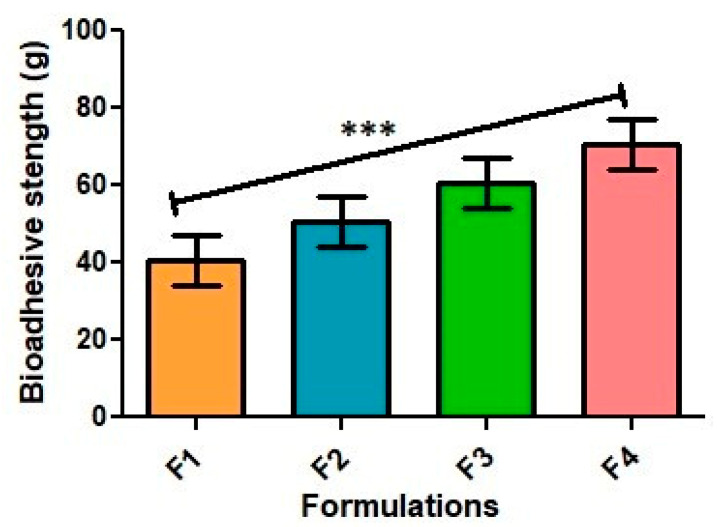
Bio-adhesive strength of all formulations (g). The results are enumerated as mean ± S.D (*n* = 3). *** represents significance level (*p* ≤ 0.001).

The bio-adhesive strength of an emulgel refers to its ability to adhere to biological surfaces such as skin, mucous membranes, or wounds. The bio-adhesive strength of an emulgel is important, because it affects the duration of contact between the emulgel and the biological surface, which can impact the effectiveness of the treatment. The bio-adhesive strength was found in the range of 46 ± 1.52 for F1 and 69.33 ± 0.88 for F4, as shown in Figure 5. The increased bio-adhesive strength of the F4 formulation refers to the increased concentration of the Carbopol and the inclusion of both natural oils. The bio-adhesive strength of an emulgel can be affected by several factors, such as the type and concentration of the bio-adhesive polymer used and the nature of the emulsifying agent (Carbopol).

### 2.6. Extrudability

Extrudability refers to the ability of a product to be easily dispensed from its packaging. In the case of an emulgel, extrudability refers to the ease with which the product can be squeezed out of a tube or bottle. Emulgels are designed to have a semi-solid, gel-like consistency that makes them easy to apply and absorb quickly into the skin. However, this consistency can also make it challenging for the product to be easily dispensed from its packaging. To address this issue, emulgel formulations may include additives such as thickeners or viscosity enhancers, which can help to improve the product’s extrudability. Some factors that can affect the extrudability of an emulgel include the product’s viscosity, the type of packaging used, and the environmental conditions in which the product is stored. For example, if an emulgel has a high viscosity, it may be more difficult to squeeze out of a tube or bottle. Similarly, if the product is stored in a cold environment, the emulgel may become more solid and difficult to extrude. The results concluded that the F4 formulation was found in the range from 68.78 ± 0.41 to 85.76 ± 0.19 (Table 2). The F4 formulation had the maximum extrudability owing to the optimum viscosity.

### 2.7. Antibacterial Studies

**Figure 6:**
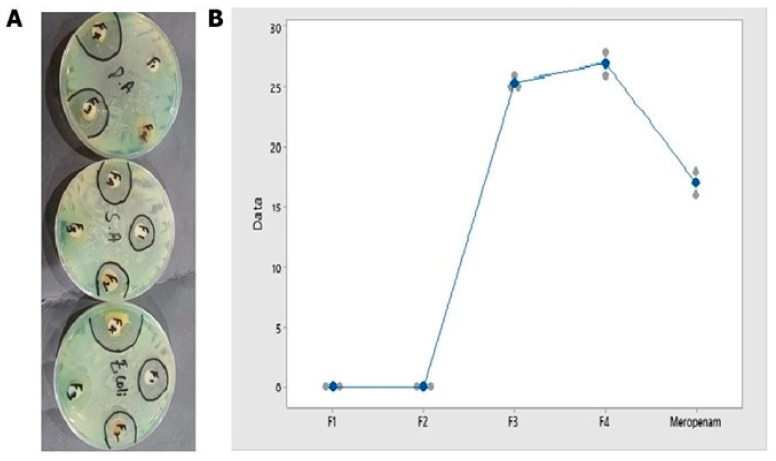
Image of Petri plates showing zones of inhibitions produced by all formulations against *Pseudo*, *Staph*, and *E. coli* (**A**), individual value plot of all formulations vs. standard Meropenem (**B**).

Antibacterial studies proved that the maximum zone of inhibition (mean ± SEM) was observed against *E. coli* produced by the extract and F4 at 41.66 ± 0.88 and 27 ± 0.5 mm, respectively (Table 3). The mean zone of inhibition produced by all the formulations against *E. coli* was found to be 24.417 ± 2.065 (mean ± S.D), which was the greatest among all. All the formulations, collectively, showed a statistically significant antibacterial effect all-tested bacterium *p*-value = 0.028 (Table 3). F1 and F2 were found to be sensitive for *Staphylococcus aureus* and *E. coli*. F3 was found to only be active against *Pseudomonas* and the F4 formulation was found to be active against all strains of bacteria. About three (75%) formulations were found to be active against *Staphylococcus* and *E. coli*, while two (50%) formulations were found to be active against *Pseudomonas*. The mean zone of inhibition produced by F4 was the greatest among all at 26.44 ± 0.37 mm (mean ± S.D). The maximum zone of inhibition was produced against *E. coli* (mean ± S.D) at 24.417 ± 2.06. The F4 formulation showed 100% activity against all microbes, *Staph*, *Pseudo*, and *E. coli* (mean ± S.D) (25.33 ± 0.28, 27.33 ± 0.5, and 27 ± 0.5), respectively (Table 4). The antibacterial activity shown by F4 was much greater in comparison to the standard, as shown in Figure 6.

### 2.8. In Vitro Dissolution Study

**Figure 7:**
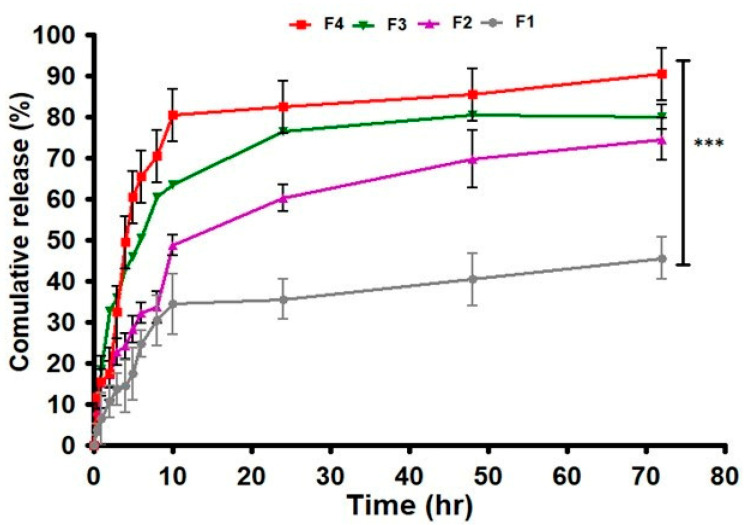
Individual value plot of % drug dissolution from F1 to F4. The results are enumerated as mean ± S.D (*n* = 3). *** represents significance level (*p* ≤ 0.001).

The in vitro dissolution study results showed that the maximum drug released from the F4 formulation through the dissolution study was reported (mean ± S.D) as 86.35 ± 0.576 with *p*-value of 0.000 (Table 5, Figure 7). F3 showed a 62% drug release, F2 showed 45%, and F1 showed 12%. Table 6 discusses the various pharmacokinetic parameters of drug release in vitro. As per our findings, a first-order model was best suited for our emulgels, as the correlation coefficient R^2^ value was close to 1 in first-order kinetics. First-order drug release is dependent upon the concentration of the drug. The F4 formulation contained olive oil, which has been used as a permeation enhancer in various topical formulations, as it has also been reported to provide excellent drug release [28].

### 2.9. Ex Vivo Permeation study

The ex vivo permeation study proved that the maximum drug release from the F4 formulation via the permeation study was reported [28] as 55.29 ± 0.12 with a *p*-value of 0.000 (Table 7). The presence of olive oil had made it a strong permeation enhancer [22].

## 3. Conclusions

Herbal emulgels containing plant extract of *Spilanthes acmella* possess strong antibacterial potential against various deep skin tissue infections which cannot be treated by antibiotics due to the emerging drug resistance caused by *Staphylococcus aureus*, *Pseudomonas*, and *E. coli*. The F4 formulation was proven to be the most effective against all the tested strains of bacteria. The F4 formulation contained olive oil, which proved to be a strong permeation enhancer; due to this reason, our optimized formulation showed wonderful responses in the in vitro and ex vivo studies. Consequently, *Spilanthes acmella* extract-based emulgel containing spilanthol would be a better alternative as a topical drug delivery system for the treatment of antibacterial problems of the skin.

## 4. Materials and Methods

### 4.1. Materials

*Spilanthes acmella* extract, lemon oil, olive oil, carbopol 935, Tween 20, liquid paraffin, methyl paraben, ethyl paraben, ethanol, and Span 20 were purchased from Sigma Aldrich, Darmstadt, Germany.

#### Method of Preparation of *Spilanthes acmella* Loaded Emulgel

The extract of *Spilanthes acmella* was prepared using an already developed method [29], and the emulgel was also prepared using the same method already reported, but with two different permeation enhancers—lemon and olive oil. As per Ansel H.C. et al. [30], the emulsion was prepared first, then it was gellified with carbapol-934 gel using an already developed method [31,32]. The composition of four batches of emulgel contained 2% *Spilanthes acmella* extract *W*/*W* (Table 8). The HLB of the lemon oil = 12 [33] and the HLB of the olive oil = 7 [34]. The HLB of liquid paraffin = 10 [35]. The values of the span and tween were calculated using the Griffin method [36]. Emulgels were characterized for parameters such as physical examination, rheological studies, spreading coefficient, bio-adhesive strength measurement, extrudability study, anti-bacterial activity, FTIR analysis, in vitro dissolution, and ex vivo permeation studies.

### 4.2. Physical Examination

The prepared batches of emulgels were examined visually for phase separation, homogeneity, consistency, grittiness, and color.

### 4.3. Fourier Transforms Infrared Spectroscopy (FT-IR)

For analyzing compatibility, FTIR studies were performed. The pure extract and all formulations were analyzed using Perkin Elmer-spectrum (Waltham, MA, USA) over the wavenumber range of 3500–1000 cm^−1^ [37].

### 4.4. Viscosity

The viscosity of all formulations was checked by using a B-One Plus viscometer (SN: 18.12.PB049, Paris, France) using spindle no. 7 at 150 rpm for durations of 10 min readings, which were performed in triplicate [38].

### 4.5. Determination of Spreading Coefficient

The spreading coefficient was determined using an already developed method [39], as per that reported by Das K et al., 2009. The following formula was used to calculate the spreading coefficient:

S = M × L/T(1)

here, S = spreadability, L = length of glass plate, W = tied weight to the upper plate, and T = time taken in seconds.

### 4.6. Bio-Adhesive Strength Measurement

For analyzing the bio-adhesive strength, the same method reported by Jones DS [40] in 1997 was used. The formula for the bio-adhesive strength calculation is given below [41,42]:

Bio-adhesive Strength = Required weight (grams)/Area (cm^2^). (2)

### 4.7. Extrudability Study of Topical Emulgel

An extrudability test was performed using collapsible aluminum tubes with the method reported by Wood JH in 1963 [43]. The formula for the calculation is given below:

(3) $$\mathrm{Extrudability}=\frac{weight\;applied\;to\;exclude\;emulgel\;outside\;tube\;(gram)}{area\;({cm}^{2})}$$

### 4.8. Antibacterial Activity

The anti-bacterial activity against three microbes, *Staphylococcus aureus* (ATCC 25923), *Escherichia coli* (ATCC 25922), and *Pseudomonas aeruginosa* (ATCC 27853), was investigated using the agar well diffusion method with an already developed method [44]. These microbial strains were collected from the Microbiology department, Bahauddin Zakariya University, Multan. The means were analyzed using a one way analysis of variance (ANOVA), followed by Tukey’s post hoc multiple comparison test using Minitab 17 software. The results were expressed as mean ± S.D. *p* values of <0.05 were considered as significant [11].

### 4.9. In Vitro Drug Release Studies

The in vitro drug release studies of all the emulgels were carried out using dialysis membrane. First, the membrane was soaked in phosphate-buffered solution at pH 7.4 for 24 h. About 2 g of emulgel containing 400 mg of extract was spread on the dialysis membrane, which was then placed on a dissolution basket. About 100 mL of (PBS) phosphate-buffered solution was added to the receptor compartment for 4 h. The whole assembly was stirred with a magnetic stirrer (Clifton, Rhine-Westphalia, Germany) and a 37 ± 0.5 °C temperature was maintained. About 10 mL of sample was withdrawn after every 30 min duration and then replaced with fresh dissolution media in an equal amount. The samples were analyzed at 229 nm by using a spectrophotometer (Waltham, MA, USA), and then the cumulative percentage of the drug release was calculated [45,46].

### 4.10. Ex Vivo Permeation Studies through Franz’s Diffusion Cell

The ex vivo drug release was analyzed on F1–F4 by using Franz diffusion cells (Lahore, Pakistan). Albino male rat skin was used, with a pH of 7.4 Phosphate buffer was placed in the receptor compartment. About a 7 mL buffer volume was used. The Franz diffusion cell temperature was maintained at 37 ± 0.1 °C with approximately a 1 cm radius of the donor compartment. In total, 7.5 mg of emulgel containing 1.5 mg of extract was applied on the rat skin. The whole assembly was stirred at 100 rpm by magnetic stirrer. The samples were withdrawn after time intervals in minutes. The duration of the study was 80 min. All the samples were evaluated at a 229 nm wavelength [47].

### 4.11. Statistical Analysis

AN ANOVA and student T-test were preferred for the statistical analysis in assessing the results and identifying the level of significance *p* ≤ 0.05.

## Figures and Tables

**Table 1:** The FTIR analysis of pure extract and formulations (F1–F4).

Pure Extract Wave no. cm^−1^	F1 Wave no. cm^−1^	F2 Wave no. cm^−1^	F3 Wave no. cm^−1^	F4 Wave no. cm^−1^
3244.76	2918.25	3381.99	3365.43	3365.51
1636.32	2875.20	2957.63	2878.96	2879.54
1403.76	2359.77	2878.96	2133.14	2123.68
1048.68	1900.71	2358.40	1638.80	1638.87
930.85	1686.99	1636.40	1402.87	1559.48
677.47	1457.52	1377.52	1330.88	1403.60
-	1373.07	1058.74	1039.68	1332.87
-	1062.79	750.82	-	1039.43
-	-	-	-	745.53

**Table 2:** Percentage extrudability of all formulations of emulgel. The results are enumerated as mean ± S.D (*n* = 3).

Formulation	Net wt. of Emulgel in Tube (g) Mean ± SEM	Wt. of Emulgel Extruded in (g) Mean ± SEM	Extrudability Amount in Percentage (%age) Mean ± SEM	Grade	
**F1**	12.97 ± 0.00	9.16 ± 0.03	70.67 ± 0.23	Fair	++
**F2**	12.67 ± 0.01	10.86 ± 0.03	85.76 ± 0.19	Good	+++
**F3**	12.50 ± 0.00	8.6 ± 0.05	68.78 ± 0.41	Fair	++
**F4**	12.15 ± 0.07	10.1 ± 0.05	83.12 ± 0.64	Good	+++

**Table 3:** Antibacterial effect of each formulation, standard, and extract. The results are enumerated as mean ± S.D (*n* = 3).

	*Staphylococcus aureus* (Mean ± SEM)	*Pseudomonas aeruginosa* (Mean ± SEM)	*E. coli* (Mean ± SEM)
F1	19.33 ± 0.28	0	23 ± 0.5
F2	22.66 ± 0.28	0	22.33 ± 0.28
F3	0	25.33 ± 0.28	0
F4	25.33 ± 0.28	27 ± 0.5	27 ± 0.5
Extract	25.33 ± 0.88	27.33 ± 0.66	41.66 ± 0.88
Meropenem	0	17 ± 0.57	16.33 ± 0.33

**Table 4:** Analysis of Variance of F1 to F4 against each bacterium (*E. coli*, *Pseudo*, and *Staph*). The results are enumerated as mean ± S.D (*n* = 3).

Source	DF	Adj SS	Adj MS	F-Value	*p*-Value
**Formulations**	3	800.1	400.03	4.00	0.028
**Error**	33	3297.5	99.92	-	-
**Total**	35	4097.6	-	-	-

**Table 5:** Analysis of Variance of F1 to F4 % age dissolution. The results are enumerated as mean ± S.D (*n* = 3).

Source	DF	Adj SS	Adj MS	F-Value	*p*-Value
**Formulations**	3	475.978	158.659	874.17	0.000
**Error**	8	1.452	0.181	-	-
**Total**	11	477.430	-	-	-

**Table 6:** Drug release linetic parameters of all formulations at 7.4 pH. The results are enumerated as mean ± S.D (*n* = 3).

Formulation	Zero Order		First Order		Higuchi with F_0_ Model		Korsmeyeres Peppas F_0_ Model			Hixon Crowell with t_lag_	
	K0	R^2^	k1	R^2^	kH	R^2^	kKP	R^2^	*n*	kHC	R^2^
**F1**	10.45	0.755	1.90	0.99	31.94	0.836	18,222.7	0.908	0.001	0.085	0.824
**F2**	13.38	0.971	0.391	0.99	37.51	0.987	58.29	0.988	0.349	0.081	0.986
**F3**	15.64	0.987	0.258	0.995	43.46	0.994	38.12	0.994	0.55	0.099	0.995
**F4**	13.85	0.913	0.989	0.983	39.92	0.954	33,633.64	0.983	0.001	0.109	0.952

**Table 7:** Mean percentage of drug released via permeation from each formulation at pH 7.4. The results are enumerated as mean ± S.D (*n* = 3).

Formulation	Mean ± St. Dev.	95% CI
**F1**	36.54 ± 0.193	(36.340, 36.747)
**F2**	44.95 ± 0.153	(44.7498, 45.1568)
**F3**	51.40 ± 0.127	(51.1965, 51.6035)
**F4**	55.29 ± 0.127	(55.0865, 55.4935)

**Table 8:** Composition of all batches of emulgels. The results are enumerated as mean ± S.D (*n* = 3).

Components	F1	F2	F3	F4
Extract	2	2	2	2
Carbopol 935	1	2	1	2
Lemon oil	7	7	-	-
Olive oil	-	-	2	2
Span 20	0.7	0.7	0.9	0.9
Tween 20	0.2	0.2	0.08	0.08
Liquid paraffin	7.5	7.5	7.5	7.5
Ethanol	2.5	2.5	2.5	2.5
Methyl paraben	0.03	0.03	0.03	0.03
Ethyl paraben	0.01	0.01	0.01	0.01
Propylene glycol	5	5	5	5
Triethanolamine	1–2 drops	1–2 drops	1–2 drops	1–2 drops
Water	q.s to 100 g	q.s to 100 g	q.s to 100 g	q.s to 100 g

## Data Availability

All data and materials are available on request from the corresponding author. The data are not publicly available due to ongoing research using a part of the data.

## References

[1] Sroka-Tomaszewska J., Trzeciak M. (2021). Molecular mechanisms of atopic dermatitis pathogenesis. Int. J. Mol. Sci..

[2] Puar N., Chovatiya R., Paller A.S. (2021). New treatments in atopic dermatitis. Ann. Allergy Asthma Immunol..

[3] Ratchataswan T., Banzon T.M., Thyssen J.P., Weidinger S., Guttman-Yassky E., Phipatanakul W. (2021). Biologics for treatment of atopic dermatitis: Current status and future prospect. J. Allergy Clin. Immunol. Pract..

[4] Ollech A., Mashiah J., Lev A., Simon A.J., Somech R., Adam E., Barzilai A., Hagin D., Greenberger S. (2021). Treatment options for DOCK8 deficiency-related severe dermatitis. J. Dermatol..

[5] Simpson E.L., Silverberg J.I., Nosbaum A., Winthrop K.L., Guttman-Yassky E., Hoffmeister K.M., Egeberg A., Valdez H., Zhang M., Farooqui S.A. (2021). Integrated safety analysis of abrocitinib for the treatment of moderate-to-severe atopic dermatitis from the phase II and phase III clinical trial program. Am. J. Clin. Dermatol..

[6] Jahan F., Lawrence R., Kumar V., Junaid M. (2011). Evaluation of antimicrobial activity of plant extracts on antibiotic susceptible and resistant *Staphylococcus aureus* strains. J. Chem. Pharm. Res..

[7] Papp K., Szepietowski J.C., Kircik L., Toth D., Eichenfield L.F., Leung D.Y., Forman S.B., Venturanza M.E., Sun K., Kuligowski M.E. (2021). Efficacy and safety of ruxolitinib cream for the treatment of atopic dermatitis: Results from 2 phase 3, randomized, double-blind studies. J. Am. Acad. Dermatol..

[8] Jansen R.K. (1985). The systematics of Acmella (Asteraceae-Heliantheae). Systematic Botany Monographs.

[9] Ramsewak R.S., Erickson A.J., Nair M.G. (1999). Bioactive N-isobutylamides from the flower buds of *Spilanthes acmella*. Phytochemistry.

[10] Chakraborty A., Devi R.K., Rita S., Sharatchandra K., Singh T.I. (2004). Preliminary studies on antiinflammatory and analgesic activities of *Spilanthes acmella* in experimental animal models. Indian J. Pharmacol..

[11] Prabuseenivasan S., Jayakumar M., Ignacimuthu S. (2006). In vitro antibacterial activity of some plant essential oils. BMC Complement. Altern. Med..

[12] Tranter H., Tassou S.C., Nychas G. (1993). The effect of the olive phenolic compound, oleuropein, on growth and enterotoxin B production by *Staphylococcus aureus*. J. Appl. Bacteriol..

[13] Newsom M., Bashyam A.M., Balogh E.A., Feldman S.R., Strowd L.C. (2020). New and emerging systemic treatments for atopic dermatitis. Drugs.

[14] Talat M., Zaman M., Khan R., Jamshaid M., Akhtar M., Mirza A.Z. (2021). Emulgel: An effective drug delivery system. Drug Dev. Ind. Pharm..

[15] Khan B.A., Ullah S., Khan M.K., Alshahrani S.M., Braga V.A. (2020). Formulation and evaluation of *Ocimum basilicum*-based emulgel for wound healing using animal model. Saudi Pharm. J..

[16] Azam F., Alqarni M.H., Alnasser S.M., Alam P., Jawaid T., Kamal M., Khan S., Alam A. (2023). Formulation, In Vitro and In Silico Evaluations of Anise (*Pimpinella anisum* L.) Essential Oil Emulgel with Improved Antimicrobial Effects. Gels.

[17] Sadeq Z.A., Sabri L.A., Al-Kinani K.K. (2022). Natural polymer Effect on gelation and rheology of ketotifen-loaded pH-sensitive in situ ocular gel (Carbapol). J. Adv. Pharm. Educ. Res..

[18] Hemalatha B., Priya T.P., Manasa K., Greeshmika C., Kavya P., Sarah S.S., Padmalatha K.J.P. (2022). Optimization of Oxiconazole Topical Emulgel Formulation for the Treatment of Skin Infections. Asian J. Pharm. Technol..

[19] Hasan S., Bhandari S., Sharma A. (2022). Formulation and Evaluation of Luliconazole Emulgel. Int. J. Res. Publ. Rev..

[20] Tranchida P.Q., Bonaccorsi I., Dugo P., Mondello L., Dugo G. (2012). Analysis of Citrus essential oils: State of the art and future perspectives. A review. Flavour Fragr. J..

[21] Al-Qudah T.S., Zahra U., Rehman R., Majeed M.I., Sadique S., Nisar S., Tahtamouni R., Tahtamouni R.W. (2018). Lemon as a source of functional and medicinal ingredient: A review. Int. J. Chem. Biochem. Sci..

[22] Kariman N. (2015). Assessing comparison the effect of cooling gel pads and topical olive oil on the intensity of episiotomy pain in primiparous women. Complement. Med. J..

[23] Cougnard-Gregoire A., Merle B.M., Korobelnik J.-F., Rougier M.-B., Delyfer M.-N., Le Goff M., Samieri C., Dartigues J.-F., Delcourt C. (2016). Olive oil consumption and age-related macular degeneration: The ALIENOR Study. PLoS ONE.

[24] Pereira A.P., Ferreira I.C., Marcelino F., Valentão P., Andrade P.B., Seabra R., Estevinho L., Bento A., Pereira J.A. (2007). Phenolic compounds and antimicrobial activity of olive (*Olea europaea* L. Cv. Cobrançosa) leaves. Molecules.

[25] Hombach M., Bloemberg G.V., Böttger E.C. (2012). Effects of clinical breakpoint changes in CLSI guidelines 2010/2011 and EUCAST guidelines 2011 on antibiotic susceptibility test reporting of Gram-negative bacilli. J. Antimicrob. Chemother..

[26] Berger-Bächi B., Barberis-Maino L., Strässle A., Kayser F.H. (1989). FemA, a host-mediated factor essential for methicillin resistance in *Staphylococcus aureus*: Molecular cloning and characterization. Mol. Gen. Genet. MGG.

[27] Mohanty D., Bakshi V., Singh M.A., Aamiruddin M., Rashaid M.A., Raj M.P., Niharika M., Reddy N.B. (2016). Formulation and Characterization of Transdermal Patches of Amlodipine Besylate Using Olive Oil as the Natural Permeation Enhancer. Am. J. Pharm. Res..

[28] Bao Q., Newman B., Wang Y., Choi S., Burgess D.J. (2018). In vitro and ex vivo correlation of drug release from ophthalmic ointments. J. Control. Release.

[29] Afzal A., Shah N.H., Hussain I., Munawar S.H., Mumtaz A., Qureshi N. (2022). Preparation of Spilanthes acmella based emulgel: Antimicrobial study and evaluation. Pak. J. Pharm. Sci..

[30] Masar B. (2004). Formulation and Evaluation of Meloxicam as a Topical Preparation. Master’s Thesis.

[31] Khullar R., Kumar D., Seth N., Saini S. (2012). Formulation and evaluation of mefenamic acid emulgel for topical delivery. Saudi Pharm. J..

[32] Mohamed M.I. (2004). Optimization of chlorphenesin emulgel formulation. AAPS J..

[33] Niczinger N., Kállai-Szabó N., Dredán J., Budai L., Hajdú M., Antal I. (2015). Application of droplet size analysis for the determination of the required HLB of lemon oil in O/W emulsion. Curr. Pharm. Anal..

[34] Yukuyama M.N., Kato E.T.M., de Araujo G.L.B., Löbenberg R., Monteiro L.M., Lourenço F.R., Bou-Chacra N.A. (2019). Olive oil nanoemulsion preparation using high-pressure homogenization and d-phase emulsification—A design space approach. J. Drug Deliv. Sci. Technol..

[35] Orafidiya L.O., Oladimeji F. (2002). Determination of the required HLB values of some essential oils. Int. J. Pharm..

[36] Pasquali R.C., Taurozzi M.P., Bregni C. (2008). Some considerations about the hydrophilic–lipophilic balance system. Int. J. Pharm..

[37] Javed H., Shah S.N.H., Iqbal F.M. (2018). Formulation development and evaluation of diphenhydramine nasal nano-emulgel. AAPS Pharmscitech.

[38] Bonacucina G., Cespi M., Palmieri G.F. (2009). Characterization and stability of emulsion gels based on acrylamide/sodium acryloyldimethyl taurate copolymer. AAPS Pharmscitech.

[39] Das K., Dang R., Machale M. (2009). Formulation and evaluation of a novel herbal gel of Stevia extract. Iran. J. Dermatol..

[40] Jones D.S., Woolfson A.D., Brown A.F. (1997). Textural, viscoelastic and mucoadhesive properties of pharmaceutical gels composed of cellulose polymers. Int. J. Pharm..

[41] Tasdighi E., Azar Z.J., Mortazavi S.A. (2012). Development and in-vitro evaluation of a contraceptive vagino-adhesive propranolol hydrochloride gel. Iran. J. Pharm. Res. IJPR.

[42] Manne N., Yadav H.K., Kumar S.H., Khom T.C., Kumar N.S. (2014). Design and evaluation of a lyophilized liposomal gel of an antiviral drug for intravaginal delivery. J. Appl. Polym. Sci..

[43] Wood J.H., Catacalos G., Lieberman S. (1963). Adaptation of commercial viscometers for special applications in pharmaceutical rheology II: Severs extrusion rheometer. J. Pharm. Sci..

[44] Arora S., Vijay S., Kumar D. (2011). Phytochemical and antimicrobial studies on the leaves of *Spilanthes acmella*. J. Chem. Pharm. Res.

[45] Khalil Y.I., Khasraghi A.H., Mohammed E.J. (2011). Preparation and evaluation of physical and, rheological properties of clotrimazole emulgel. Iraqi J. Pharm. Sci..

[46] Varma V.N.S.K., Maheshwari P., Navya M., Reddy S.C., Shivakumar H., Gowda D.J. (2014). Calcipotriol delivery into the skin as emulgel for effective permeation. Saudi Pharm. J..

[47] Chang J., Zhao Y., Zhao W., Venkataramanan R., Caritis S.N. (2014). Obstetrical-Fetal Pharmacology Research Units Network. Quality assessment of compounded 17-hydroxyprogesterone caproate. Am. J. Obstet. Gynecol..

